# Growing-finishing performance and carcass yield of pigs reared in a climate-controlled and uncontrolled environment

**DOI:** 10.1007/s00484-014-0908-3

**Published:** 2014-11-19

**Authors:** Mariana Piatto Berton, Rita de Cássia Dourado, Flávia Biondi Fernandes de Lima, Ana Beatriz Bertoncello Rodrigues, Fábio Borba Ferrari, Leonardo Dimas do Carmo Vieira, Pedro Alves de Souza, Hirasilva Borba

**Affiliations:** Department of Technology, College of Agricultural and Veterinary Sciences, Sao Paulo State University, Via de Acesso Prof. Paulo Donato Castellane s/n, 14884-900 São Paulo, Brazil

**Keywords:** Environment, Loin eye area, Feed conversion, Thermoneutrality

## Abstract

The objective of this study was to evaluate the effect of temperature on the performance and carcass yield of pigs housed in different environments. Twenty castrated male pigs of the Topigs line were assigned to two treatments: T1, controlled environment, and T2, uncontrolled environment containing a shallow pool. A completely randomized design consisting of two treatments and ten repetitions each was used. The data were submitted to analysis of variance, and means were compared by the Tukey test at a level of significance of 5 % using the SAS 9.2 program (SAS Institute, Inc., NC, USA). The results showed that rearing pigs in an uncontrolled environment during the growing and finishing phases reduced daily feed intake (1.722 and 3.164 kg, respectively) and improved feed conversion (2.15 and 2.70 kg, respectively), but did not influence the carcass yield of the animals. In conclusion, rearing pigs under different environmental conditions during the growing and finishing phases influenced animal performance without interfering with carcass yield.

## Introduction

Pork is the most widely consumed meat in the world, and Brazil is the world’s fourth largest producer of pork, with a production of approximately 3.483 million tons in 2012 (USDA 2006; ABIPECS 2012). In an attempt to better attend the demand and to increase the productivity of production systems, pork producers have used new technologies to mitigate some of the factors that can limit production, such as animal nutrition, physiology, and health.

As homeothermic animals, pigs possess an internal thermoregulatory system which is triggered in the case of unfavorable environmental conditions. When pigs are submitted to an environment whose temperature is lower than the body temperature, heat dissipates from their body to the environment. Taking the physical laws of heat transfer as a basis, this is a normal process in which the system has a tendency to reach an equilibrium state. These situations are perceived by peripheral thermoreceptors (cells located on the skin) and analyzed by neural mechanisms that make an adequate decision and activate specific agents. Pigs adapt to hot or cold environments and are highly sensitive to adverse climatic conditions in both cold and hot climates.

In cold climates, newborn piglets are more affected because of their deficient thermoregulatory control. In contrast, in hot climates, adult animals are more affected since, as they acquire a greater subcutaneous fat layer, they become unable to dissipate body heat. The amount of heat exchanged by the animal with the environment decreases with increasing temperature until a minimum equilibrium is reached (Ferreira 2000).

It is known that the environment influences animal performance, i.e., in the absence of an adequate environment, the animal is unable to show its maximum genetic potential, to maintain its health (perfect health status) and to nourish itself properly in terms of the consumption and utilization of nutrients since energy is redirected for the maintenance of body temperature. The maintenance of the animal’s comfort is therefore fundamental to ensure good performance of pigs (Sarubbi 2005) and to prevent the animals from becoming less efficient in the utilization of available energy (Kerr et al. 2003).

External environmental factors and the microclimate inside the facilities exert direct and indirect effects on pigs during all stages of production; however, these effects are aggravated during the final stages of production, including an increase in the sensitivity of pigs to heat and a reduction in productivity, with consequent economic losses for pig farming (Hannas 1999).

According to Ferreira (2005), the effects of external environmental factors on performance are more drastic during the growing and finishing phases when compared to other stages of production due to the increase in subcutaneous fat deposition which, in turn, impairs the dissipation of heat produced during metabolic processes. The consequences of any reduction in feed intake, either due to a heat wave or poorly designed ventilation, are rapidly noted since reduced feed intake leads to a decrease in the intake of nutrients, thus affecting performance. However, worsening in the feed conversion rate may not be observed since there is a decrease in feed intake and in weight gain.

Many alternatives can be adopted by producers in order to reduce the effects of the environment on pigs and to maximize productivity, such as the correct position of facilities, tree planting around the facilities, the use of fans and reflecting tiles, semi-climatized pens, and the use of shallow pools (Moreira et al. 2003).

The objective of the present study was to evaluate the effect of temperature on the performance and carcass yield of pigs housed in different environments.

## Material and methods

This experiment was conducted in the pig farming sector of the School of Agricultural and Veterinary Sciences (Faculdade de Ciências Agrárias e Veterinárias, FCAV/UNESP), Jaboticabal, São Paulo, Brazil. Twenty castrated male pigs of the Topigs line acquired after weaning, aged approximately 21 days and weighing 23 kg, were randomly assigned to the treatments and reared until reaching a body weight of approximately 100 kg when they were slaughtered. During the growing and finishing phases, the animals were fed with the corn-soybean meal-based diet described in Table 1 formulated according to the recommendations of Rostagno et al. (2005) in order to meet the nutritional requirements of each phase. The diet was offered ad libitum and was replaced twice a day.

**Table 1:** Nutritional requirements of castrated male pigs with a high genetic potential and regular performance

Phase	Growing				Finishing	
Live weight (kg)	30 to 50	50 to 70			70 to 100	
Feed intake (kg/day)	1.963	2.680			3.197	
Digestible lysine requirement (g/day)	14.981	18.793			19.439	
Nutrient						
Metabolizable energy (Kcal/kg)	3230	3230			3230	
Protein (%)	15.80	14.30			12.71	
Calcium (%)	0.631	0.551			0.484	
Total phosphorus (%)	0.524	0.459			0.412	
Available phosphorus (%)	0.332	0.282			0.248	
Potassium (%)	0.448	0.425			0.400	
Sodium (%)	0.180	0.170			0.160	
Chloride (%)	0.170	0.160			0.150	
Amino acid	Dig	Total	Dig	Total	Dig	Total
Lysine (%)	0.758	0.861	0.696	0.692	0.609	0.692
Methionine (%)	0.227	0.250	0.209	0.229	0.189	0.208
Methionine + cystine (%)	0.455	0.508	0.418	0.467	0.378	0.422
Tryptophan (%)	0.136	0.155	0.125	0.143	0.116	0.132
Threonine (%)	0.493	0.594	0.452	0.546	0.408	0.491
Arginine (%)	0.311	0.336	0.285	0.308	0.185	0.208
Valine (%)	0.523	0.603	0.480	0.554	0.420	0.484
Isoleucine (%)	0.417	0.474	0.383	0.435	0.335	0.381
Leucine (%)	0.758	0.836	0.696	0.767	0.609	0.671
Histidine (%)	0.250	0.276	0.230	0.253	0.201	0.221
Phenylalanine (%)	0.379	0.422	0.348	0.388	0.305	0.339
Phenylalanine + tyrosine (%)	0.758	0.844	0.696	0.775	0.609	0.678

The nutrient percentage was determined using digestible lysine requirements according to performance, amino acid/lysine ratio, and equation of nutrient/Mcal.ME. Total lysine requirement was calculated considering the average true digestibility of lysine to be 88 %

The following treatments were evaluated: treatment 1, controlled environment characterized by a constant temperature of 22 °C and relative humidity of 70 %; treatment 2, uncontrolled environment in which the animals were subjected to climatic changes of the environment using a shallow pool.

For control of the environment in treatment 1, air conditioners (Split Springer, with a cooling capacity of 18,000 BTUs), which were automatically controlled by thermostats and turned on when the ambient temperature exceeded 22 °C, and climate-control units (Hidromotor HM250, with an air flow rate of 12,000 m^3^/H), were used. Two exhaust fans (Ventisol, with an air flow rate of 5000 m^3^/h, power of 1/4 cv and rpm of 1400, 50/60 Hz), measuring 50 cm in diameter, were installed for oxygen renewal of the environment in order to prevent the influence of ammonia released from the excretions of the animals on their respiration. The room was also equipped with recording devices for maximum and minimum temperature and relative air humidity, which were installed at half the height of the animal’s body. The temperature and humidity were measured twice a day in the morning and afternoon throughout the experiment. The animals were housed in pairs in pens with a concrete floor, measuring 2.0 × 3.0 m (height 3.2 m), equipped with feeders and nipple drinkers. The animals were separated from each other by grids that allowed them to see each other.

The animals of treatment 2 were housed in pairs in pens with a concrete floor, measuring 1.5 × 4.0 m (height 3.2 m), equipped with feeders and nipple drinkers. The animals were separated from each other by grids that allowed them to see each other. A shallow pool (1 m wide and 10 cm deep) was constructed on the lower side of the inclined floor.

For the determination of feed intake, the ration offered and the leftovers were weighed at intervals of 3 days during the growing (up to 60 kg) and finishing phases (60 to 100 kg). The animals were weighed weekly. Average daily weight gain was obtained by subtracting the final from the initial weight divided by the number of days of rearing. Feed conversion was calculated by dividing feed intake by weight gain. Mortality was observed throughout the experimental period and during the pre-slaughter phase, evaluating factors that can influence the mortality of animals such as time of fasting and transport.

The animals were weighed before slaughter to obtain the body weight at slaughter (SW) and fasted from solid food and water for 16 h at the School of Animal Science and Food Engineering, University of São Paulo (Faculdade de Zootecnia e Engenharia de Alimentos da Universidade de São Paulo, FZEA/USP), Pirassununga, São Paulo. At 6 a.m., the animals were transported to the slaughterhouse in a simple truck after a 12-h fast, receiving only water. For slaughter, the animals were submitted to electric stunning at 220 ± 20 V and a frequency of 60 Hz.

After cleaning, the carcasses were weighed for the determination of hot carcass weight (HCW) and cold carcass weight (CCW) was measured after 24 h in the cooling chamber (0 to 2 °C). Carcass yield (CYCCW/SW× 100) and weight loss due to cooling (WL[(HCW − CCW/HCW) × 100]) were then calculated.

The carcass measures were obtained on the slaughter line. Carcass length was measured with a graded metal tape measure from the first rib to the pubic symphysis according to the Brazilian method of carcass evaluation (ABCS 1973). Backfat thickness was measured at the height of the first rib (BF1), last rib (BF2), last lumbar vertebra (BF3), and maximum lumbar vertebra with a digital caliper. For the measurement of backfat depth and loin eye area, the tenth rib was first located as described by Silveira (2007). The loin eye area was measured by delimiting the contour of the longissimus dorsi muscle wrapped in plastic with a special pen and calculating the area using a planimeter.

A completely randomized design consisting of two treatments and ten repetitions each was used. The data were submitted to analysis of variance, and means were compared by the Tukey test at a level of significance of 5 % using the SAS 9.2 program (SAS Institute, Inc., NC, USA).

## Results

**Fig. 1:**
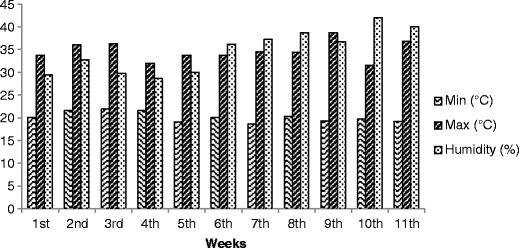
Variation of minimum and maximum temperatures recorded and the relative air humidity in the uncontrolled environment throughout the experiment

The temperature and relative humidity of the controlled environment were maintained at 22 °C and 70 %, respectively, and the mean minimum and maximum recorded temperatures and the relative air humidity of the uncontrolled environment were 20 °C, 34.5 °C, and 36 % respectively, throughout the experimental period. The variation of the temperature and humidity in the treatment 2, during the experiment, is presented in Fig. 1.

Table 2 shows the mean daily feed intake, weight gain, and feed conversion of pigs during the growing and finishing phases.

**Table 2:** Daily feed intake (FI), weight gain (WG), and feed conversion (FC) during the growing and finishing phases

Treatment	Trait					
	Growing			Finishing		
	FI	WG	FC	FI	WG	FC
Treatment 1	2.158A	0.806	2.677 A	3.660 A	1.135	3.224 A
Treatment 2	1.722 B	0.801	2.149 B	3.164 B	1.172	2.699 B
*P* value	<0.001	0.926	<0.001	<0.001	0.516	<0.001
CV (%)	7.97	4.46	9.84	4.46	13.03	9.946

Means in the same column followed by different letters differ significantly by the Tukey test (5 %)

*Treatment 1* controlled environment, *treatment 2* uncontrolled environment, *CV* coefficient of variation

A significant difference in feed intake and feed conversion was observed between treatments during the growing and finishing phases. Animals submitted to climatic variations (reared in a non-climate-controlled facility, treatment 2) showed lower feed intake (*P* < 0.05) and worse feed conversion (*P* < 0.05) during the growing and finishing phases, whereas weight gain was not significantly affected (*P* > 0.05) by the treatments. These findings suggest that animals maintained at ambient temperature in the presence of a shallow pool obtained better performance than those reared in a controlled environment.

Table 3 shows the mean HCW, CCW, CY, and WL. No significant difference (*P* > 0.05) was observed between treatments. This finding might be explained by the presence of the shallow pool, which may have alleviated the high temperatures reached in the environment, exerting no influence on weight or CY.

**Table 3:** Mean hot carcass weight (HCW), cold carcass weight (CCW), carcass yield (CY), and weight loss due to cooling (WL)

Treatment	Trait			
	HCW	CCW	CY	WL
Treatment 1	85.04	83.38	73.58	1.95
Treatment 2	85.25	83.55	73.54	2.0
*P* value	0.913	0.929	0.723	0.723
CV (%)	5.0	5.0	0.29	14.61

Means in the same column followed by different letters differ significantly by the Tukey test (5 %)

*Treatment 1* controlled environment, *treatment 2* uncontrolled environment, *CV* coefficient of variation

Table 4 shows the mean carcass length, backfat depth, loin eye area, backfat thickness measured at three sites in the carcass (BF1, BF2, and BF3), and average backfat thickness.

**Table 4:** Mean carcass length (CL), backfat depth (BD), loin eye area (LEA), backfat thickness (BF1, BF2 and BF3), and average backfat thickness (ABF)

Treatment	Trait						
	CL	BD	LEA	BF1	BF2	BF3	ABF
Treatment 1	95.95	60.5	46.30	41.08	29.07	25.44	31.86
Treatment 2	96.00	59.7	43.00	42.14	31.25	28.3	33.89
*P* value	0.977	0.782	2.59 NS	0.687	0.108	0.232	0.224
CV (%)	4.06	9.73	10.26	13.9	9.51	19.26	10.97

*Treatment 1* controlled environment, *treatment 2* uncontrolled environment, *CV* coefficient of variation, *NS* not significant

No significant differences in the carcass traits studied (*P* > 0.05) were observed between treatments. The animals of the present study did not differ in terms of weight gain or carcass traits. This finding might be due to the presence of a shallow pool for animals maintained at ambient temperature, which alleviated the effect of thermal stress and prevented the animals from spending production energy for thermal maintenance.

## Discussion

The consequences of a reduction in feed intake, either due to a heat wave or poorly designed ventilation, are rapidly noted since reduced feed intake leads to a decrease in the intake of nutrients, thus affecting performance. However, worsening in the feed conversion rate may not be observed since there is a decrease in feed intake and in weight gain (Ferreira 2005). Studies investigating the response of growing pigs kept at different temperatures concluded that heat stress causes behavioral disturbances and negatively affects performance (feed intake, weight gain, and feed conversion; (Kiefer et al. 2009)).

Moreira et al. (2003), evaluating the performance of pigs (33 to 84 kg) reared in pens with a compact floor and shallow pool, observed similar performance responses for the different treatments. In a study on the effects of ambient temperature on the performance of pigs weighing 30 to 60 kg, Manno et al. (2006) reported better feed conversion for animals reared in the high-temperature environment compared to those maintained in thermal comfort. However, the authors observed no difference in carcass yield or carcass weight between the different treatments in agreement with the results of the present study (Table 3).

The results showed in Table 3 were similar to those reported by Collin et al. (2001) who, studying piglets weighing 24 to 30 kg, demonstrated better feed efficiency for animals exposed to a high temperature compared to those maintained in thermal comfort. According to these authors, pigs kept under heat stress are metabolically more efficient in synthetic processes, reducing protein turnover. The most likely reason for the turnover of body protein is the need for fine-tuning amino acid and protein metabolism (Buttery 2013) in addition to guaranteeing homeothermy in mammals (Lobley 2003).

The feed intake of animals reared at ambient temperature in the presence of a shallow pool was lower than that of animals reared in the controlled environment (*P* < 0.05). The same was observed by Dourmand and Noblet (1998) who, studying the effect of ambient temperature on feed intake in pigs, observed a 17 % decrease in feed intake when the temperature increased from 20 to 28 °C. This fact can be explained by the mechanisms that regulate body temperature in homeothermic animals. Pigs consume less food at high ambient temperature and increase consumption when the temperature decreases. According to Quiniou et al. (2000) and Le Bellego et al. (2002), the reduction in feed intake observed in pigs submitted to high ambient temperatures is probably a defense mechanism of the organism to reduce heat production resulting from digestive and metabolic processes.

Tavares et al. (2000) evaluated the influence of ambient temperature on the performance of pigs and found that, under heat stress, the animals gained less weight, but carcass traits were not affected. Moreira et al. (2003), studying the performance of pigs (33 to 84 kg) reared in pens with a compact floor or shallow pool, observed no difference in backfat thickness between treatments in agreement with the present results (Table 4).

The amplitude of the thermal environment inside the facilities can disturb the thermodynamic defense mechanism of animals, with repercussions on zootechnical indices (Ferreira 2005). Pigs maintained in a controlled environment tend to express their maximum genetic potential; however, when the effective ambient temperature increases, the animals use behavioral, physical, and chemical mechanisms that can lead to redirection of the energy available for production, modifying the nutritional requirements of the animals (Orlando et al. 2001).

## Conclusions

The shallow pool provides a comfortable thermal environment similar to the controlled environment and favors animal performance. Under the present experimental conditions, the different environments did not affect the carcass traits of pigs.

## References

[CR1] ABCS - Associação Brasileira De Criadores De Suínos (1973) Método brasileira de classificação de carcaça. Estrela. 17p. Publicação técnica, n. 2

[CR2] ABIPECS. Associação Brasileira da Indústria Produtora e Exportadora de Carne (2012) http://www.abipecs.org.br/pt/estatisticas.html. Accessed 12 August 2013

[CR3] Buttery PJ (2013) Protein turnover in animals. Tropical animal production, Merida, Mexico, v. 6, n. 3, p. 204–213, 1981. http://www.fao.org/ag/aga/agap/frg/tap63/63_204.pdf. Accessed 12 August 2013

[CR4] Collin A, VAN Milgen J, Dubois S, Noblet J (2001). Effect of high temperature and feeding level on energy utilization in piglets. J Anim Sci, Savoy.

[CR5] Dourmand JY, Noblet J (1998) Genetics, environment, and nutrition interrelationships in swine production. In: Simpósio Sobre Nutrição Animal e Tecnologia da Produção de Rações, São Paulo. Anais… Campinas: CBNA, 1998. p. 155–168

[CR6] Ferreira RA (2000) Efeitos do clima sobre a nutrição de suínos. In: Encontros Técnicos da Abraves, 2000, Chapecó. Memórias… Concórdia: ABRAVES, v. 1, p. 1–15

[CR7] Ferreira RA (2005). Maior produção com melhor ambiente para aves, suínos e bovinos.

[CR8] Hannas MI, Silva IJO (1999). Aspectos fisiológicos e a produção de suínos em clima quente. Ambiência e qualidade na produção industrial de suínos.

[CR9] Kerr BJ, Yen JT, Nienaber JA, Easter RA (2003). Influences of dietary protein level, amino acid supplementation and environment temperature on performance, body composition, organ weights and total heat production of growing pigs. J Anim Sci, Savoy.

[CR10] Kiefer C, Meignen BCG, Sanches JF, Carrijo AS (2009). Resposta de suínos em crescimento mantidos em diferentes temperaturas. Arch Zootec, Cordoba, Spain.

[CR11] LE Bellego I, Van Milgen J, Noblet J (2002). Effect of high temperature and low-protein diets on the performance of growing-finishing pigs. J Anim Sci, Savoy.

[CR12] Lobley GE (2003). Protein turnover—what does it mean for animal production?. Can J Anim Sci.

[CR13] Manno MC, Oliveira RF, Donzele JL (2006). Efeitos da temperatura ambiente sobre o desempenho de suínos dos 30 aos 60 kg. Rev Bras Zootec.

[CR14] Moreira I, Paiano D, de Oliveira GC, Gonçalves GS, Neves CA, Barbosa OR (2003). Desempenho e características de carcaça de suínos (33–84 kg) criados em baias de piso compacto ou com lâmina d′água. Rev Bras Zootec, Viçosa, MG.

[CR15] Orlando UAD, Oliveira RFM, Donzele JL (2001). Níveis de proteína bruta da ração para leitoas dos 30 aos 60kg mantidas em ambiente de conforto térmico (21 °C). Rev Bras Zootec, Viçosa, MG.

[CR16] Quiniou N, Dubois S, Noblet J (2000). Voluntary feed intake and feeding behavior of grouphoused growing pigs are affected by ambient temperature and body weight. Livest Prod Sci, Amsterdam.

[CR17] Rostagno HS, Albino LFT, Donzele JL, Gomes PC, de Oliveira RF, Lopes DC, Ferreira AS, Barreto SL de T (2005) Tabelas brasileiras para aves e suínos: composição de alimentos e exigências nutricionais. 2nd ed. Viçosa, MG: Editora UFV, 186 p

[CR18] Sarubbi J (2005) Estudo do conforto térmico, desempenho animal e racionalização de energia em uma instalação de suínos na região de Boituva – SP. 2005 120f. Dissertação (Mestrado em Engenharia Agrícola) – Universidade Estadual de Campinas

[CR19] Silveira ETF (2007) Inovações tecnológicas aplicadas na determinação da composição da carcaça e suas implicações na industrialização da carne suína. In: Seminário de aves e suínos, 7., 2007, Belo Horizonte. Anais… [Belo Horizonte]: AveSui. p. 96–109

[CR20] Tavares SLS, Donzele JL, Oliveira RFM, Ferreira AS (2000) Influence of environment temperature on the performance and physiological traits of barrows farm 30 to 60kg. Revista Brasileira de Zootecnia. v.29, n.1, p. 199–205

[CR21] U.S. Department of Agriculture (2006) Composition of foods, raw, processed, prepared USDA National Nutrient Database for Standard Reference, release Nutrient Data Laboratory homepage. Maryland. http://www.nal.usda.gov/fnic/foodcomp/Data. Accessed 11 February 2013

